# SNP rs3803264 polymorphisms in *THSD1* and abnormally expressed mRNA are associated with hemorrhagic stroke

**DOI:** 10.3389/fnagi.2023.1144364

**Published:** 2023-04-17

**Authors:** Changying Chen, Xincheng Gu, Fangyuan Liu, Congyong Sun, Jialin Mu, Defu Jin, Xuemei Sui, Deqin Geng, Qingqing Li, Yuzhang Jiang, Chong Shen

**Affiliations:** ^1^Department of Epidemiology, Center of Global Health, School of Public Health, Nanjing Medical University, Nanjing, Jiangsu, China; ^2^Department of Medical Laboratory, Huai’an First People’s Hospital, The Affiliated Huai’an No.1 People’s Hospital of Nanjing Medical University, Huai’an, Jiangsu, China; ^3^Department of Neurology, The Affiliated Hospital of Xuzhou Medical University, Xuzhou, Jiangsu, China; ^4^Department of Neurology, The Third People's Hospital of Xuzhou, Xuzhou, Jiangsu, China

**Keywords:** thrombospondin type 1 domain containing 1, hemorrhagic stroke, case–control study, single nucleotide polymorphisms, mRNA expression

## Abstract

**Background:**

Thrombospondin Type 1 Domain Containing Protein 1 (THSD1) has been suggested to be a new regulator of endothelial barrier function in the angiogenesis process, preserving vascular integrity. We sought to characterize the association of *THSD1* genetic variants and mRNA expression with the risk of hemorrhagic stroke (HS) with population-based evidence.

**Methods:**

A case–control study was conducted with 843 HS cases and 1,400 healthy controls. A cohort study enrolled 4,080 participants free of stroke at baseline in 2009 and followed up to 2022. A synonymous variant, the main tag SNP rs3803264 of the *THSD1* gene, was genotyped in all subjects, and peripheral leukocyte *THSD1* mRNA expression was detected using RT-qPCR in 57 HS cases and 119 controls.

**Results:**

In the case–control study, rs3803264 AG/GG variations are associated with a decreased risk of HS with odd ratio (*OR*) and 95% confidence interval (*CI*) of the dominant model of 0.788 (0.648–0.958), *p* = 0.017. In addition, rs3803264 and dyslipidemia had a multiplicative interaction [*OR* (95% *CI*) = 1.389 (1.032, 1.869), *p* = 0.030]. In the cohort study, a similar association strength of rs3803264 dominant model and the risk of HS was observed with the incidence rate ratio (*IRR*) of 0.734 and *p*-value of 0.383. Furthermore, the risk of HS showed a non-linear as *THSD1* mRNA expression increased (*p* for non-linearity <0.001). For the subjects without hypertension, we observed *THSD1* mRNA expression had a negative correlation with systolic blood pressure (SBP; *ρ* = −0.334, *p* = 0.022).

**Conclusion:**

SNP rs3803264 polymorphisms in *THSD1* are associated with the decreased risk of HS and interacted with dyslipidemia, and a non-linear association was observed between *THSD1* mRNA expression and the risk of HS.

## Introduction

Though hemorrhagic stroke (HS) has a lower incidence rate, it is associated with severe morbidity, high mortality, and worse outcomes (Unnithan et al., 2022). HS often appears as severe neurological dysfunction with underlying vascular causes, such as sudden vascular rupture or occlusion, which is attributed to an acute focal neurological injury (Unnithan et al., 2022). As reported, a history of hypertension, diabetes, dyslipidemia, smoking, and alcohol abuse was considered as the modifiable risk factors for HS (Feigin et al., 2016; Pandian et al., 2018). In the vast majority of cases, genetic risk variants contribute to a multifactorial predisposition to stroke (Montaner et al., 2020). To date, previous genome-wide association studies (GWAS) and candidate gene association studies have identified 42 loci that are robustly associated with stroke, but only 3 loci were associated with intracerebral hemorrhage (ICH) and predominantly observed in European ancestry populations (Woo et al., 2014; Neurology Working Group of the Cohorts for Heart and Aging Research in Genomic Epidemiology (CHARGE) Consortium, the Stroke Genetics Network (SiGN), and the International Stroke Genetics Consortium (ISGC), 2016; Malik et al., 2018). Therefore, statistical associations of specific loci with HS need to be complemented to improve our understanding of the molecular pathways that underlie HS and identify novel drug targets.

Thrombospondin type I domain-containing protein 1 (THSD1) is a transmembrane protein whose function remains poorly characterized (Takayanagi et al., 2006). In 2010, the tumor-suppressive and angiogenic role of *THSD1* in esophageal carcinoma cells was reported (Ko et al., 2008), and *THSD1* is a critical tumor-suppressive region in esophageal carcinoma (Khamas et al., 2012). The common pathological processes of angiogenesis in tumor enlightened the researchers that whether *THSD1* also play a significant role in intracerebral hemorrhage. Teresa et al. demonstrated that *THSD1* is mainly expressed in endothelial cells in murine cerebral arteries (Santiago-Sim et al., 2016). *THSD1* has been linked to vascular permeability (Shamseldin et al., 2015). Recently, it has been proposed that *THSD1* has a potential role in angiogenesis and maintenance of vascular integrity (Dekker et al., 2011). Furthermore, rs3803264 at *THSD1* is involved in angiogenesis, apoptosis, and activation of transforming growth factor beta (*TGFβ*; Lavrov et al., 2016). It is a surface marker of hematopoietic progenitors and endothelial cells (Takayanagi et al., 2006). Collectively, *THSD1* has been suggested to be a new regulator of endothelial barrier function in the angiogenesis process, preserving vascular integrity, which may be involved in the pathophysiology of HS.

Previous studies primarily focused on the roles *THSD1* played in the angiogenesis process, the association between *THSD1* with HS still needs to elucidate. Meanwhile, changes in mRNA levels in HS have the potential to aid HS diagnosis and provide insight into stroke etiology (Montaner et al., 2020). Therefore, we conducted a case–control and cohort study to investigate the association of *THSD1* genetic variants and mRNA expression with the risk of hemorrhage stroke. This epidemiological study would provide a population-based genomic and transcriptomic level evidence for better understanding of the role *THSD1* in the pathogeny of HS.

## Materials and methods

### Study subjects in the case–control study and cohort study

A hospital-based case–control study was conducted on consecutive patients recruited from three hospitals in Jiangsu province from Sep 2008 to Jun 2021, including the Affiliated Huai’an NO.1 People’s Hospital of Nanjing Medical University, the Affiliated Hospital of Xuzhou Medical University, and the Affiliated Yixing Hospital of Jiangsu University. The study population consisted of 843 hemorrhagic stroke (HS) patients and 1,400 healthy controls enrolled from a community-based population who underwent health examination without hemorrhagic or ischemic stroke history. All subjects were of Chinese Han descent. The exclusion and inclusion criteria for patients and controls were similar to our previous study (Shen et al., 2013). HS was confirmed by brain computed tomography (CT) or magnetic resonance imaging (MRI) scans in all HS patients. We divided HS patients into intracerebral hemorrhage (ICH) and subarachnoid hemorrhage (SAH). Primary intraventricular hemorrhage and cerebral hemorrhage caused by trauma, hemorrhagic transformation of infarction, cerebrovascular abnormalities, brain tumors, aneurysms, or hemorrhagic diatheses due to antithrombotic therapy were excluded in this study. Additionally, for the mRNA expression study, the mRNA isolated from peripheral blood leukocytes was compared between 57 HS patients from three hospitals in Jiangsu Province and gender and age-matched 119 controls from community surveys. The demographic and clinical characteristics of the study subjects in the above two parts were listed in Table 1.

**Table 1:** Demographic and clinical characteristics of the subjects in the case–control study.

Characteristics	Group	Total population				Subgroups for mRNA comparison			
		Control (*n* = 1,400)	HS (*n* = 843)	*Z/χ^2^*	Value of *p*	Control (*n* = 119)	HS (*n* = 57)	*Z/χ^2^*	Value of *p*
Age (year) M (IQR)		64.9 (58.3, 71.8)	59.0 (50.0, 69.0)	9.585	<0.001^a^	65.0 (55.0, 73.0)	59.0 (51.0, 69.5)	1.699	0.089^a^
Gender [*n* (%)]	Male	630 (45.0)	492 (58.4)	37.583	<0.001^b^	81 (68.1)	37 (64.9)	0.174	0.677^b^	Female	770 (55.0)	351 (41.6)			38 (31.9)	20 (35.1)		
	SBP (mmHg) M (IQR)		135 (124, 150)	154 (139, 170)	16.255	<0.001^a^	140 (128, 156)	163 (146, 179)	5.229	<0.001^a^
DBP (mmHg) M (IQR)		81 (73, 89)	90 (80, 100)	15.506	<0.001^a^	84 (78, 94)	92 (84, 103)	4.000	<0.001^a^
GLU (mmol/L) M (IQR)		5.94 (5.25, 6.11)	6.30 (5.21, 7.71)	7.423	<0.001^a^	5.69 (5.34, 6.56)	5.73 (5.05, 7.81)	0.305	0.760^a^
TC (mmol/L) M (IQR)		5.17 (4.53, 5.75)	4.06 (3.30, 4.80)	21.384	<0.001^a^	4.66 (4.03, 5.32)	4.31 (3.69, 5.17)	1.386	0.166^a^
TG (mmol/L) M (IQR)		1.36 (0.95, 1.87)	1.20 (0.81, 1.74)	5.106	<0.001^a^	1.46 (0.91, 2.11)	1.14 (0.74, 1.49)	2.596	0.009^a^
HDL-C (mmol/L) M (IQR)		1.40 (1.20, 1.57)	1.15 (0.85, 1.33)	16.924	<0.001^a^	1.11 (0.91, 1.38)	1.16 (0.98, 1.41)	0.711	0.477^a^
LDL-C (mmol/L) M (IQR)		2.29 (1.90, 2.85)	2.31 (1.77, 2.83)	1.368	0.171^a^	2.69 (2.14, 3.07)	2.63 (1.90, 3.27)	0.077	0.938^a^
Hypertension [*n* (%)]	Yes	747 (53.4)	747 (88.6)	294.031	<0.001^b^	76 (63.9)	53 (93.0)	16.693	<0.001^b^	No	653 (46.6)	96 (11.4)			43 (36.1)	4 (7.0)		
	Diabetes [*n* (%)]	Yes	284 (20.3)	314 (37.3)	77.425	<0.001^b^	29 (24.4)	21 (36.8)	2.948	0.086^b^	No	1,116 (79.7)	529 (62.8)			90 (75.6)	36 (63.2)		
Dyslipidemia [*n* (%)]		Yes	923 (65.9)	512 (60.7)	6.157	0.013^b^	69 (58.0)	31 (54.4)	0.203	0.652^b^	No	477 (34.1)	331 (39.3)			50 (42.0)	26 (45.6)		

HS, hemorrhagic stroke; SBP, systolic blood pressure; DBP, diastolic blood pressure; GLU, glucose; TC, total cholesterol; TG, triglyceride; HDL-C, high-density lipoprotein-cholesterol; LDL-C, low-density lipoprotein-cholesterol.

a Mann–Whitney *U*-test.

b *χ*^2^ test.

**Figure 1:**
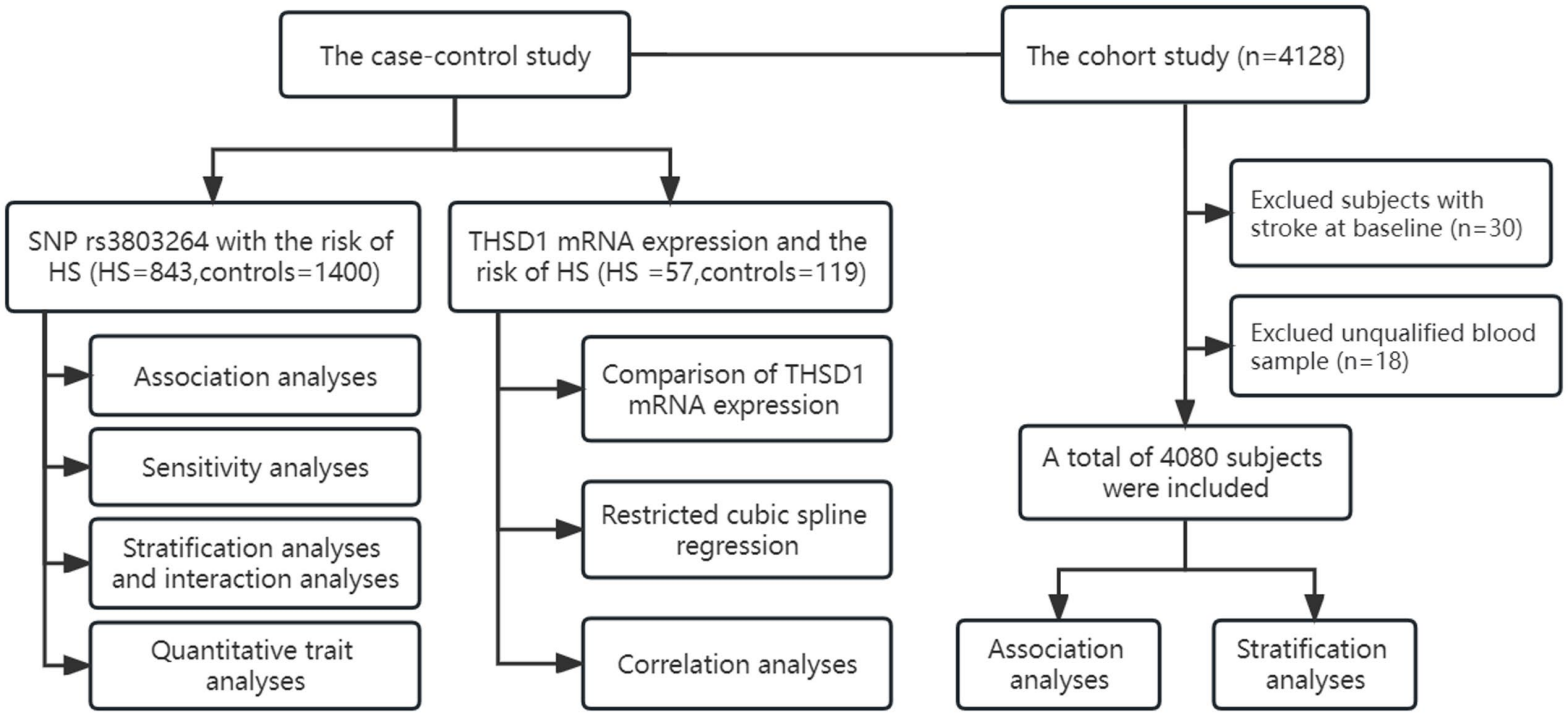
The flow chart of study design and statistical analyses. We conducted a case–control study and a cohort study to investigate the association of *THSD1* genetic variants and mRNA expression with the risk of hemorrhage stroke.

The community-based cohort study was conducted from 2009 to 2022 and recruited 4,128 participants from Guanlin Town and Xushe Town, Yixing city (Jiangsu, China). 4,080 baseline subjects without stroke and strictly controlled were followed up until May 25, 2022, for stroke onset. Detailed information about this cohort has been described previously (Dong et al., 2021). Demographic and clinical characteristics of the study population in the cohort study were summarized in Supplementary Table S2. The flow chart of this study was shown in Figure 1.

### Questionnaire survey, physical examination, and biochemical index detection

Epidemiological information was recorded by questionnaires, and demographic information (gender and age), clinical characteristics (blood pressure, blood glucose levels, and plasma lipids), and history of the disease (hypertension, diabetes, and dyslipidemia) were collected. The patient’s blood pressure was measured within 48 h of hospitalization after the onset of HS symptoms. Peripheral venous blood was collected after an overnight fast (>10 h) for analyzing the fasting glucose and plasma lipid levels, including total cholesterol (TC), triglycerides (TG), low-density lipoprotein cholesterol (LDL-C), and higher-density lipoprotein cholesterol (HDL-C). Specifically, hypertension was defined as definite hypertension history, or elevated levels of measured blood pressure [systolic blood pressure (SBP) ≥140 mmHg, diastolic blood pressure (DBP) ≥90 mmHg], or under antihypertensive treatment. Diabetes was defined as definite diabetes history, fasting blood glucose level (GLU) ≥ 7.0 mmol/L, or under hypoglycemic treatment. Dyslipidemia was defined as definite dyslipidemia history, or abnormal changes in lipid levels (TC ≥ 6.2 mmol/L, TG ≥ 2.3 mmol/L, LDL-C ≥ 4.1 mmol/L, HDL-C < 1.04 mmol/L), or under lipid-modulating therapy. The details of blood sample collection and the methods of biochemical index detection were described previously (Chen et al., 2022).

### Single nucleotide polymorphisms selection and genotyping

The *THSD1* gene (Gene ID: 55901) is located on chromosome 13 at q14.3 (from 52,951,302 to 52,980,307) with 29,006 nt. We searched the SNPs from the upstream 2 kb to the downstream 1 kb and selected tagging SNPs (tagSNPs) through the database of the Chinese Han population in Beijing (CHB) and China of the International Hap MAP Project. Two tagSNPs (tagSNPs rs3803264 and tagSNP rs9563101) would be available for candidate SNP selection. Included tagSNPs met the criteria of minor allele frequency (MAF) ≥ 0.05, linkage disequilibrium (LD) *r*^2^ ≥ 0.8, and probes and primers designed successfully. The probe and primer designing section is known as an *in silico* procedure performed in dry lab (Behzadi and Ranjbar, 2019). Finally, only rs3803264 (A > G) was selected and genotyped in this study, because the probes and primers rs9563101 were not designed successfully and the MAF of rs9563101 in East Asian is 0.05. Detailed biological information and function prediction were summarized in Supplementary Table S1.

The details of DNA extraction and preservation and genotyping were described previously (Chen et al., 2022). The polymerase chain reaction (PCR)-TaqMan MGB probe array was performed for the genotyping of *THSD1* polymorphism. The forward primer sequence was 5′-ACCTTCCAAGTGGGCCTATTTAC-3′, the reverse primer sequence was 5′-GGAAGACTGTTGGTGAAGATGACA-3′, and the probes sequences of rs3803264 were 5′-AAGCCCAACATTGTAGTG-3′ and 5′-AAGCCCAACATCGTA-3′. The genotyping successful call rate of rs3803264 was 100%. The amplification products of rs3803264 were sequenced and validated (Supplementary Figure 1).

### RNA extraction, reverse transcription, and quantitative real-time polymerase chain reaction

The methods and reagents of white blood cell separation and preservation, RNA extraction, reverse transcription, and quantitative real-time polymerase chain reaction (RT-qPCR) were consistent with the previous study (Chen et al., 2022). All samples were analyzed in three parallels, and cycle threshold values were recorded. The 2 ^−ΔΔCT^ method was used to calculate relative expression levels of *THSD1* normalized by glyceraldehyde-3-phosphate dehydrogenase (*GAPDH*). *THSD1* mRNA’s forward primer sequence (5′-3′) was TGTGACTATGTTCTTGGAGAAGC, and the reverse primer sequence (5′-3′) was GTGTCCCATTAGCACCATCAAAA. *GAPDH* mRNA’s forward primer sequence (5′-3′) was GGAGCGAGATCCCTCCAAAAT, and the reverse primer sequence (5′-3′) was GGCTGTTGTCATACTTCTCATGG. The amplification products of *THSD1* were sequenced and validated (Supplementary Figure 2).

### Statistical analysis

We used EpiData 3.1 software (The EpiData Association, Odense, Denmark) for duplicate entry and consistency checks of the collected data. Continuous variables were presented as median [inter-quartile range (IQR)] for nonparametric data. Categorical variables were presented as frequencies and percentages. For group-wise comparisons, the Kruskal–Wallis test or Mann–Whitney test was used for continuous variables with an abnormal distribution. The chi-square test (*χ*^2^) was used to compare the differences in categorical variables between case and control groups. Fisher’s exact test was used to estimate whether the genotype frequencies in the controls and HS group met the Hardy–Weinberg equilibrium (HWE) law. Binary logistic regression was applied to calculate the odds ratios (*OR*s) and corresponding 95% confidence intervals (*CI*s) for the association of *THSD1* variants and hypertension and HS. Stratification analyses by age group, gender, hypertension, diabetes, and dyslipidemia were further conducted in the case–control study. Inter-subgroup heterogeneity among the stratum factors was assessed using the Heterogeneity Q test. The interaction of dyslipidemia and *THSD1* rs3803264 polymorphism with HS was estimated. The multiplicative interaction hazard ratio was calculated by fitting the logistic regression model. The additive interaction was displayed by calculating the relative excess risk due to interaction (RERI), attributable proportion (AP), synergy index (SI), and 95% *CI*s. We used Poisson regression to estimate the association with incidence rate ratios (*IRR*s) and 95% *CI*s as well as after adjustment for covariates in the cohort study. The kernel density estimation graph was conducted to show the distribution of *THSD1* mRNA expression. We also used restricted cubic splines (RCS) with four knots at the 20th, 40th, 60th, and 80th centiles to flexibly model the association of *THSD1* mRNA expression with the risk of HS. We used Spearman’s rank correlation to evaluate the correlations between *THSD1* mRNA expression and clinical indicators. All data analyses were carried out using SAS software 9.4 (SAS Inc., Cary, NC, United States) and R 4.1.1 version (http://cran.r-project.org/). A two-tailed *p*-value <0.05 was considered statistically significant.

## Results

### Demographic and clinical characteristics of the study subjects

Table 1 shows the detailed demographic and clinical characteristics of 843 HS cases and 1,400 controls in the genetic case–control study. The median age of the HS group (59 years) was significantly younger than that of the control group (65 years, *p* < 0.001). HS group had higher proportions of males (58.4%) than the control group (45.0%; *p* < 0.001). There were significant differences in SBP, DBP, GLU, TC, TG, and HDL-C levels (*p* < 0.001) except LDL-C levels (*p* = 0.171) between HS cases and controls. HS group had a higher prevalence of hypertension (88.6%) and diabetes (37.3%) while a lower prevalence of dyslipidemia (60.7%) than controls (53.4, 20.3, and 65.9%; *p* < 0.05).

In the case–control study for transcriptome level analysis, age, and gender were both matched for the HS case and control. HS group presented a higher level of SBP and DBP but lower levels of TG than the control group (Table 1). HS group had a higher prevalence of hypertension (93.0%) than the control group (63.9%; *p* < 0.001). The demographic and clinical characteristics of the cohort study for the incidence of HS were presented in Supplementary Table S2.

### Association analyses of *THSD1* rs3803264 with the risk of hemorrhagic stroke

The genotype and allele distributions of rs3803264 were consistent with the *HWE* in the control and HS groups (all *p* > 0.1). Considering HS is closely related to hypertension, we analyzed the association of rs3803264 with the risk of hypertension firstly, there was no significant association (Supplementary Table S3), even after adjustment for covariates [Adjusted *OR*s (95%*CI*s) for the three genetic models: 0.997 (0.864–1.151), 0.974 (0.799–1.186), and 1.048 (0.783–1.403), respectively] (Table 2). *THSD1* rs3803264 AG/GG carriers had a significantly decreased risk of HS (Supplementary Table S3), even after adjustment for covariates (Table 2). The adjusted *OR*s (95%*CI*s) for the additive model and dominant model was 0.848 (0.734–0.979) and 0.788 (0.648–0.958). No significant association was observed for the recessive models of rs3803264 with HS (Supplementary Table S3; Table 2). Furthermore, we found rs3803264 AG/GG carriers had a reduced risk of ICH rather than SAH (Supplementary Table S3), the adjusted *OR*s (95%*CI*s) for the additive model and dominant model of rs3803264 with ICH were 0.834 (0.712–0.976) and 0.779 (0.629–0.964) (Table 2).

**Table 2:** Association analyses of rs3803264 and the risk of hypertension and hemorrhagic stroke in the case–control study.

Phenotype	Group	AA/AG/GG	*OR (95% CI)*, *p*			Allele			*p* for HWE
			Additive model	Dominant model	Recessive model	Major/Minor	*OR (95% CI)*	Value of *p*^c^	
Hypertension	Control	298/355/96	Reference	Reference	Reference	0.635/0.365	Reference		0.542	Case	637/675/182	0.997 (0.864–1.151), 0.970^a^	0.974 (0.799–1.186), 0.790^a^	1.048 (0.783–1.403), 0.752^a^	0.691/0.309	0.927 (0.814–1.055)	0.249	0.877
HS	Control	555/664/181	Reference	Reference	Reference	0.634/0.366	Reference		0.422	HS	380/366/97	0.848 (0.734–0.979), 0.024^b^	0.788 (0.648–0.958), 0.017^b^	0.857 (0.637–1.151), 0.304^b^	0.668/0.332	0.860 (0.757–0.977)	0.020	0.535	ICH	302/287/75	0.834 (0.712–0.976), 0.024^b^	0.779 (0.629–0.964), 0.022^b^	0.819 (0.592–1.134), 0.229^b^	0.671/0.329	0.848 (0.739–0.973)	0.019	0.586	SAH	78/79/22	0.840 (0.657–1.073), 0.163^b^	0.767 (0.551–1.067), 0.115^b^	0.878 (0.537–1.437), 0.606^b^	0.656/0.344	0.905 (0.718–1.141)	0.397	0.773

HS, hemorrhagic stroke; ICH, intracerebral hemorrhage; SAH, subarachnoid hemorrhage; HWE, Hardy–Weinberg equilibrium.

a Adjusted for age, gender, hemorrhagic stroke, diabetes, and dyslipidemia.

b Adjusted for age, gender, hypertension, diabetes, and dyslipidemia.

c *p*-value of *χ*^2^ test for comparison of allele frequencies between the case and control groups.

### Sensitivity analysis for the association of *THSD1* rs3803264 with the risk of hemorrhagic stroke

Considering the age and gender between HS cases and controls were significantly different, we used tendency score matching to make HS cases (*n* = 737) and controls (*n* = 719) well-matched for the sensitivity analysis. We found that the association of rs3803264 and the risk of HS were stably significant, similar to what we observed before (Supplementary Tables S4–S5).

### Stratification analyses and interaction analyses

Stratified analyses by dyslipidemia showed that *THSD1* rs3803264 AG/GG carriers had a significantly decreased risk of HS in the non-dyslipidemia group and normal lipid groups of TC, TG, HDL-C, and LDL-C [Adjusted *OR*s (95%*CI*s): 0.579 (0.422–0.793), 0.664 (0.522–0.843), 0.772 (0.614–0.970), 0.739 (0.591–0.925), and 0.721 (0.586–0.888), respectively]. In addition, there was heterogeneity between dyslipidemia groups, TC groups, and LDL-C groups (*I*^2^: 81.2, 72.9, and 63.0%, respectively; *p*-values: 0.021, 0.055, and 0.100, respectively; Table 3).

**Table 3:** Stratification analyses of dyslipidemia for the association of rs3803264 and the risk of hemorrhagic stroke in the case–control study.

Factor	Stratum	Group	AA/AG/GG	*OR (95% CI)*					
				Unadjusted dominant model	*I* ^2^	Value of *p*^b^	Adjusted dominant model^a^	*I* ^2^	Value of *p*^b^
Dyslipidemia	No	Control	173/236/68	0.573 (0.431–0.761)	88.2%	0.004	0.579 (0.422–0.793)	81.2%	0.021
		HS	165/128/38	*p =* 1.26 × 10^−4^			*p =* 0.001		
	Yes	Control	382/428/113	0.975 (0.784–1.214)			0.934 (0.726–1.202)		
		HS	215/238/59	*p =* 0.824			*p =* 0.594		
TC	<5.2 mmol/L	Control	268/350/99	0.687 (0.556–0.849)	76.2%	0.040	0.664 (0.522–0.843)	72.9%	0.055
		HS	330/302/78	*p =* 0.001			*p =* 0.001		
	≥5.2 mmol/L	Control	287/314/82	1.203 (0.821–1.763)			1.198 (0.786–1.827)		
		HS	50/64/19	*p =* 0.343			*p =* 0.400		
TG	<1.7 mmol/L	Control	363/466/128	0.753 (0.613–0.925)	0.0%	0.373	0.772 (0.614–0.970)	0.0%	0.969
		HS	276/267/73	*p =* 0.007			*p* = 0.026		
	≥1.7 mmol/L	Control	192/198/53	0.905 (0.656–1.248)			0.765 (0.519–1.127)		
		HS	104/99/24	*p =* 0.542			*p =* 0.175		
HDL-C	≥1.0 mmol/L	Control	518/615/167	0.759 (0.619–0.930)	0.0%	0.861	0.739 (0.591–0.925)	0.0%	0.710
		HS	248/218/66	*p =* 0.008			*p =* 0.008		
	<1.0 mmol/L	Control	37/49/14	0.796 (0.501–1.267)			0.828 (0.499–1.375)		
		HS	132/148/31	*p =* 0.336			*p =* 0.466		
LDL-C	<3.4 mmol/L	Control	494/601/157	0.740 (0.616–0.889)	66.0%	0.086	0.721 (0.586–0.888)	63.0%	0.100
		HS	61/63/24	*p =* 0.001			*p =* 0.002		
	≥3.4 mmol/L	Control	346/312/81	1.444 (0.854–2.439)			1.496 (0.840–2.663)		
		HS	34/54/16	*p =* 0.170			*p =* 0.171		

TC, total cholesterol; TG, triglyceride; HDL-C, high-density lipoprotein-cholesterol; LDL-C, low-density lipoprotein-cholesterol.

a Adjusted for age, gender, hypertension, diabetes.

b *p*-value for heterogeneity test.

Furthermore, we observed *THSD1* rs3803264 significantly interacted with dyslipidemia in the risk of HS. The multiplicative interaction analyses indicated that *THSD1* rs3803264 and dyslipidemia had a positive interaction, specifically in the high level of TC and LDL-C groups. The *OR* (95% *CI*) after adjustment for age, gender, hypertension, and diabetes was 1.389 (1.032, 1.869), 1.799 (1.117, 2.896), and 2.095 (1.132, 3.878), respectively. However, there was no significant addictive interaction observed (Table 4).

**Table 4:** Interaction analysis of *THSD1* rs3803264 and dyslipidemia with the risk of hemorrhagic stroke in the case–control study.

Interactive item	Interaction type	Estimates (95% CI)	Model 1	Model 2
Dyslipidemia * rs3803264	Multiplicative interaction	*OR* (95% *CI*), *p*	1.388 (1.064, 1.809), 0.015	1.389 (1.032, 1.869), 0.030
	Additive interaction	*RERI* (95% *CI*)	−0.132 (−0.330, 0.066)	−0.118 (−0.315, 0.080)
		*AP* (95% *CI*)	−0.116 (−0.241, 0.009)	−0.102 (−0.226, 0.021)
		*S* (95% *CI*)	0.510 (0.239, 1.088)	0.564 (0.272, 1.168)
		*p*	0.192	0.243
High level of TC * rs3803264	Multiplicative interaction	*OR* (95% *CI*), *p*	1.750 (1.131, 2.709), 0.012	1.799 (1.117, 2.896), 0.016
	Additive interaction	*RERI* (95% *CI*)	−0.143 (−0.388, 0.102)	−0.124 (−0.362, 0.113)
		*AP* (95% *CI*)	−0.095 (−0.240, 0.050)	−0.085 (−0.229, 0.058)
		*S* (95% *CI*)	0.780 (0.525, 1.160)	0.786 (0.511, 1.207)
		*p*	0.252	0.305
High level of TG * rs3803264	Multiplicative interaction	*OR* (95% *CI*), *p*	1.202 (0.820, 1.760), 0.345	1.026 (0.666, 1.580), 0.970
	Additive interaction	*RERI* (95% *CI*)	−0.043 (−0.241, 0.154)	0.011 (−0.189, 0.211)
		*AP* (95% *CI*)	−0.039 (−0.179, 0.101)	0.009 (−0.125, 0.144)
		*S* (95% *CI*)	0.715 (0.172, 2.975)	1.074 (0.280, 4.119)
		*p*	0.668	0.915
Low level of HDL-C * rs3803264	Multiplicative interaction	*OR* (95% *CI*), *p*	1.050 (0.633, 1.743), 0.851	1.111 (0.635, 1.942), 0.712
	Additive interaction	*RERI* (95% *CI*)	−0.047 (−0.212, 0.118)	−0.046 (−0.217, 0.124)
		*AP* (95% *CI*)	−0.069 (−0.229, 0.092)	−0.065 (−0.227, 0.098)
		*S* (95% *CI*)	1.176 (0.643, 2.150)	1.196 (0.590, 2.427)
		*p*	0.575	0.594
High level of LDL-C * rs3803264	Multiplicative interaction	*OR* (95% *CI*), *p*	1.950 (1.119, 3.399), 0.018	2.095 (1.132, 3.878), 0.018
	Additive interaction	*RERI* (95% *CI*)	−0.160 (−0.439, 0.119)	−0.161 (−0.448, 0.126)
		*AP* (95% *CI*)	−0.165 (−0.408, 0.078)	−0.162 (−0.407, 0.082)
		*p*	0.261	0.272

TC, total cholesterol; TG, triglyceride; HDL-C, high-density lipoprotein-cholesterol; LDL-C, low-density lipoprotein-cholesterol. RERI, relative excess risk due to interaction; AP, attributable proportion due to interaction; SI, synergy index. Model 1 did not adjust any covariates. Model 2 adjusted for age, gender, hypertension, and diabetes.

### Association analyses of *THSD1* rs3803264 with the incidence risk of hypertension and hemorrhagic stroke in the cohort study

After an average of 10.30-year follow-up for 2,102 subjects free of hypertension at baseline, 667 incident hypertension (31.73%) were observed and the incidence density was 394.49 (per 10^4^ person-year). The variant of rs3803264 was not associated with the incident risk of hypertension, even after adjustment for covariates [Adjusted *IRR*s (95%*CI*s) for the three genetic models: 1.078 (0.964–1.204), 1.074 (0.920–1.254), and 1.163 (0.936–1.446), respectively] (Table 5).

**Table 5:** Association analyses of *THSD1* rs3803264 with the incidence risk of hypertension and hemorrhagic stroke in the cohort study.

Outcome	Genotype	Incident cases	Person-years	Incidence density (/10^4^ Person-years)	*IRR* (95% *CI*)			*IRR* (95% *CI*)^a^^,^^b^		
					Additive model	Dominant model	Recessive model	Additive model	Dominant model	Recessive model
Hypertension	AA	271	21906.20	123.71	1.086	1.079	1.188	1.078	1.074	1.163
	AG	301	23301.55	129.18	(0.972–1.214)	(0.925–1.260)	(0.956–1.476)	(0.964–1.204)	(0.920–1.254)	(0.936–1.446)
	GG	95	6709.20	141.60	*p* = 0.143	*p* = 0.334	*p* = 0.552	*p* = 0.186	*p* = 0.368	*p* = 0.174
Hemorrhagic stroke	AA	16	21906.20	7.30	0.775	0.730	0.697	0.784	0.734	0.731
	AG	13	23301.55	5.58	(0.455–1.319)	(0.365–1.460)	(0.212–2.288)	(0.458–1.340)	(0.366–1.470)	(0.222–2.406)
	GG	3	6709.20	4.47	*p* = 0.348	*p* = 0.373	*p* = 0.552	*P* = 0.373	*p* = 0.383	*p* = 0.606

a Hypertension, Adjusted for age, gender, smoking, drinking, stroke, diabetes, and dyslipidemia.

b Hemorrhagic stroke, Adjusted for age, gender, smoking, drinking, hypertension, diabetes, and dyslipidemia.

Additionally, after an average of 12.72-year follow-up for 4,080 subjects free of stroke at baseline, 32 incident HS cases (0.78%) were observed and the incidence density was 17.35 (per 10^4^ person-year). The AG/GG carriers presented a decreased incident risk of HS but the association did not reach the statistical significance [Adjusted *IRR*s (95%*CI*s) for the three genetic models: 0.784 (0.458–1.340), 0.734 (0.366–1.470), and 0.731 (0.222–2.406), respectively] (Table 5). Furthermore, there was no significant association between rs3803264 and the risk of HS, and no remarkable heterogeneity among different dyslipidemia groups was observed in the cohort study (Supplementary Table S6). Next, we grouped the subjects into the AA group and AG + GG group to explore the association between dyslipidemia and the incidence of HS. No significant association between dyslipidemia and the incidence of HS and no remarkable heterogeneity between the AA group and AG + GG group was observed in the cohort study (Supplementary Table S7).

### Quantitative trait analyses among genotypes of rs3803264

We performed quantitative trait analyses to investigate the association among the genotypes of rs3803264 and quantitative clinical characteristics using the Kruskal-Wallis H test, results are summarized in Supplementary Table S8. No significant difference in quantitative clinical characteristics (SBP, DBP, GLU, TC, TG, HDL-C, and LDL-C) among genotypes of rs3803264 was observed both in HS cases and controls (all *p* > 0.05).

### *THSD1* mRNA expression and the risk of hemorrhagic stroke

**Figure 2:**
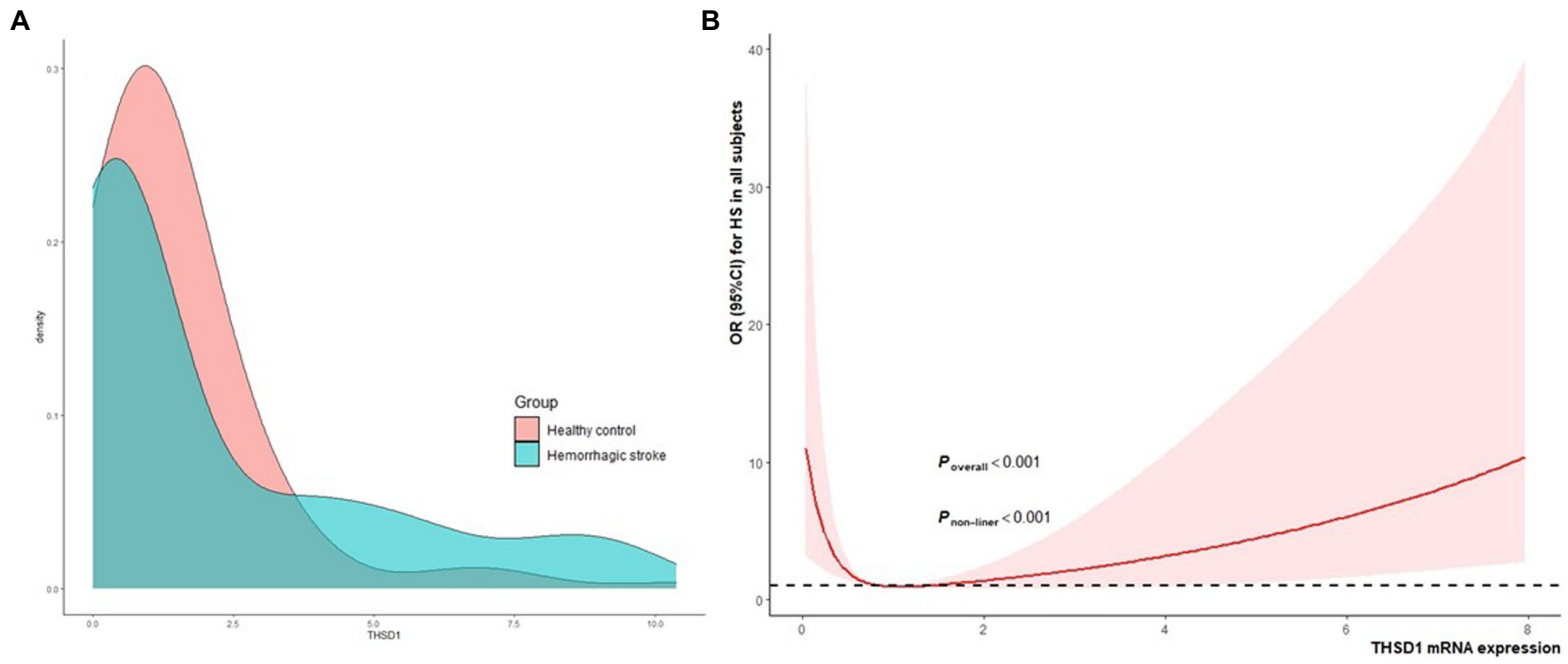
The kernel density estimation distribution of *THSD1* mRNA and the association between *THSD1* mRNA expression **(A)**, and the risk of HS in the total population was evaluated by the restricted cubic spline regression **(B)**. Estimates adjusted for age, gender, hypertension, diabetes, and dyslipidemia. The *p*-value for overall association and the *p*-value for non-linearity were annotated in the figure.

In Figure 2A, the kernel density estimation graph showed the distribution of *THSD1* mRNA expression conformed to a gamma distribution (*D* = 0.296, *p* < 0.001). *THSD1* mRNA expression was down-regulated below around 3.75, but it was upregulated above around 3.75. In addition, we used restricted cubic splines to visualize the relation of predicted *THSD1* mRNA expression with the risk of HS. In the total population (Figure 2B), the risk of HS plunged until around the median 0.878 of *THSD1* mRNA expression and then started to increase gradually afterward (*p* for non-linearity <0.001).

### Comparisons of *THSD1* mRNA expression between cases and controls

The *THSD1* mRNA expression level of HT was approximately equal to that in controls (0.88 vs. 0.86, *p* = 0.664). Besides, there was also no significant difference in *THSD1* mRNA expression between HS cases and controls (0.67 vs. 0.97, *p* = 0.571). Additionally, *THSD1* mRNA expression among HS, HT, and controls did not have statistical differences (0.67 vs. 1.03 vs. 0.85, *p* = 0.823). Additionally, no significant difference in *THSD1* mRNA expression was detected among the genotypes of rs3803264 in HS cases (*p* = 0.594) and controls (*p* = 0.416; Supplementary Figure 3A), and between HS cases and controls among the AA (*p* = 0.914), AG (*p* = 0.901) and GG (*p* = 0.115) genotype of rs3803264 (Supplementary Figure 3B).

### Correlation analyses of *THSD1* mRNA expression with clinical indicators

There was no significant linear correlation of *THSD1* mRNA expression with clinical indicators (GLU, TC, TG, HDL-C, LDL-C, SBP, and DBP) in all subjects, controls, or HS cases, no matter whether they have hypertension or not (all *p* > 0.05; Table 6). For the subjects without hypertension, we observed *THSD1* mRNA expression had a negative correlation with SBP (*ρ* = −0.334, *p* = 0.022). While for the subjects with hypertension, there was no significant linear correlation, either (all *p* > 0.05; Table 6).

**Table 6:** Correlation analyses between *THSD1* mRNA expression (2^−ΔΔCT^) and clinical indicators.

Group (*n*)	GLU	TC	TG	HDL-C	LDL-C	SBP	DBP
All subjects (176)	0.096	0.042	−0.073	0.041	0.049	−0.060	0.011
HT subjects (129)	0.138	0.063	−0.010	0.011	0.062	0.006	0.085
Non-HT subjects (47)	−0.074	−0.044	−0.251	0.153	−0.017	−0.334^*^	−0.245
All controls (119)	0.088	−0.006	−0.092	−0.031	0.042	0.002	0.043
HT (76)	0.127	0.040	−0.022	−0.100	0.079	−0.260	0.114
Non-HT (43)	−0.029	−0.099	−0.232	0.070	−0.046	0.059	−0.192
HS cases (57)	0.063	0.116	−0.024	0.122	0.110	−0.021	0.012
HT (53)	0.081	0.092	−0.020	0.099	0.082	0.067	0.095
Non-HT (4)	−0.800	0.400	<0.001	0.200	0.400	−0.800	−0.800

^*^p < 0.05; GLU, glucose; TC, total cholesterol; TG, triglyceride; HDL-C, high density lipoprotein-cholesterol; LDL-C, low density lipoprotein-cholesterol; SBP, systolic blood pressure; DBP, diastolic blood pressure; HS, hemorrhagic stroke; HT, hypertension.

## Discussion

In this study, we investigated the association of polymorphism and mRNA expression of *THSD1* with the risk of HS. In the case–control study, we found AG/GG carriers of rs3803264 had a decreased risk of HS and ICH, but not hypertension and SAH. Furthermore, the *THSD1* rs3803264 variant had significantly associated with the reduced risk of HS in subgroups of age less than or equal to 65 years, males, hypertension, non-diabetes, and non-dyslipidemia. In addition, *THSD1* rs3803264 and dyslipidemia had a positive interaction. In the cohort study, a similar association strength of rs3803264 dominant model and the risk of HS was observed but did not reach statistical significance. Referring to the mRNA level, the risk of HS dropped until around 0.878 of *THSD1* mRNA expression and then started to increase gradually afterward. For the subjects without hypertension, we observed *THSD1* mRNA expression had a negative correlation with SBP. Thus, our results demonstrated that tag rs3803264 of *THSD1* polymorphism was associated with the decreased risk of HS and interacted with dyslipidemia, and a non-linear association was observed between *THSD1* mRNA expression and the risk of HS.

HS refers to intracerebral hemorrhage and subarachnoid hemorrhage caused by intracranial aneurysms, cerebral and spinal vascular malformations, moyamoya disease, and other intracranial vascular diseases under the effect of blood flow (Unnithan et al., 2022). Intracranial aneurysm is the most common cause of subarachnoid hemorrhage (85%; Wang et al., 2017). The rupture of weakened cerebral arteries caused blood leakage in the brain, attributed to the blood extravasation and compression of the surrounding brain tissue, eventually, necrosis and apoptosis of brain cells lead to the disordered function of the central nervous system (CNS; Unnithan et al., 2022). Specifically, there are two phases in the pathological mechanism of HS, primary injuries and secondary injuries. In the primary injuries phase, hematoma causes the occupying effect and mechanical injuries to the adjacent brain tissue. Meanwhile, neurotoxins from hematoma such as hemoglobin, iron ions, reactive oxygen (ROS), and neuroexcitatory toxin initiate severe secondary injuries (Wilkinson et al., 2018). In the secondary injuries, biochemical and cellular responses to the neurotoxins from hematoma begin, such as ROS damage (Chen et al., 2011), inflammatory immune response (Ye et al., 2018), microglial polarization (Zhang et al., 2017), autophagy (Li et al., 2018), ferroptosis (Alim et al., 2019), and blood–brain barrier (BBB) collapse (Keep et al., 2018). However, irrespective of diversified mechanisms, cerebrovascular integrity is the most critical to the pathological process of HS. Particularly, the loss of vascular integrity can cause the initial hemorrhage, the failure of restoration contributes to the BBB disruption, brain edema formed, then neurotoxins from hematoma and blood cause detrimental injuries (Keep et al., 2012, 2014).

Previous studies demonstrated that *THSD1* promotes advanced lesion stability by retaining vascular integrity of the intimal neovasculature (Dekker et al., 2011). *THSD1* variants or gene loss probably cause intracranial aneurysms (IA) and SAH (Santiago-Sim et al., 2016). *THSD1* is a novel nascent adhesion protein that co-localizes with several known markers such as focal adhesion kinase (FAK), talin, and vinculin (Rui et al., 2017). Genetic evidence suggested a role of *THSD1* variants in the pathogenesis of IA/SAH, previously, and missense variants were identified in affected individuals clustered in the intracellular protein portion (Santiago-Sim et al., 2016). In addition, *THSD1* loss may lead to arterial aneurysm formation and ultimately SAH *via* the disruption of endothelial cell adhesion to the extracellular matrix in cerebral arteries potentially (Rui et al., 2017). Furthermore, *THSD1* has the effect of preserving vascular integrity in mice models, and *THSD1* is a new regulator of endothelial barrier function during vascular development and protects intraplaque microvessels against hemorrhaging in advanced atherosclerotic lesions (Haasdijk et al., 2016). Mechanistically, *THSD1* loss impaired endothelial cell focal adhesion to the basement membrane. All the above studies support the results of our study based on the population case–control study and cohort study. A recent study also supports the role of variants in *THSD1* as susceptibility factors for cerebrovascular disease (Sauvigny et al., 2020). Meanwhile, our study demonstrated that rs3803264 had a significantly decreased risk of HS in those who were younger (age less than or equal to 65 years), males, with hypertension, non-diabetes, and non-dyslipidemia. Explainable reasons include that most HS patients in this study are male, the median age is <65 years old, and most of them are hypertensive. *THSD1* rs3803264 and dyslipidemia had a positive interaction, specifically high levels of TC and LDL-C. A systematic review and meta-analysis revealed that TC is inversely associated with the risk of HS, and a higher level of LDL-C seems to be associated with a lower risk of HS (Wang et al., 2013).

In our study, the *THSD1* mRNA expression of peripheral leukocytes showed a significant non-linear association with the risk of HS, which plunged until around 0.878 and then started to increase gradually afterward. Preceding research found that *THSD1* expression by endothelial cells was detected in advanced atherosclerotic lesions with intraplaque hemorrhaging, but was not in stable lesions (Haasdijk et al., 2016). In addition, the current study identified *THSD1* down-regulation and methylation in primary colorectal cancer (CRC) tissues (Khamas et al., 2012). In our study, no significant difference in *THSD1* mRNA expression was detected among the genotypes of rs3803264 in HS cases, this result demonstrated that rs3803264 may further influence the pathogenesis of HS through post-transcriptional modification, methylation, or protein functional regulation but not mRNA expression. While for all subjects without hypertension, we observed *THSD1* mRNA expression had a negative correlation with SBP. In other words, in non-HT subjects, the lower the *THSD1* expression, the higher the blood pressure level, and the increased risk of HS. This effect warrants a further larger sample size study to validate.

This study is unique since we integrated analyses of the role *THSD1* played in HS based on the genomic level and the transcriptomic level. Moreover, we explored the association of *THSD1* candidate SNP with HS by combining the case–control study and the cohort study. In addition, the large sample size and the experiments with high-quality control ensured the accuracy and reliability of our results. However, some limitations are also worth mentioning. First, we did not detect serum THSD1 protein. Second, we selected candidate SNP at the *THSD1* gene with the criterion of MAF ≥ 0.05, so could have missed the rare variants with MAF <0.05 that may have substantial biological effects on the HS occurrence. Third, retrospective and prospective studies with a large sample size would be warranted to validate the association.

In conclusion, our results indicated that tag rs3803264 of *THSD1* polymorphism was associated with the decreased risk of HS, and the *THSD1* mRNA expression of peripheral leukocytes showed a significant non-linear association with the risk of HS. Our findings might contribute to verifying that the *THSD1* genetic variant and mRNA expression would affect the susceptibility to HS, and may provide useful evidence and guidance regarding the correlation between *THSD1* and HS.

## Data availability statement

The original contributions presented in the study are included in the article/Supplementary material, further inquiries can be directed to the corresponding authors.

## Ethics statement

All participants signed the informed consent voluntarily. The case-control and cohort study were all approved by the Research Ethics Committee of Nanjing Medical University (#200803307, #2018571) and the Medical Ethics Committee of the Affiliated Hospital of Xuzhou Medical University (#XYFY2018-KL078).

## Author contributions

ChS and YJ designed the study and edited and proofed the manuscript. CC performed the experiment work, analyzed the data, and wrote the manuscript. XG analyzed the data and proofed the manuscript. FL performed the experiment work and proofed the manuscript. CoS, DG, and QL collected the samples. DJ and XS collected the data. JM performed the experiment work. All authors contributed to the article and approved the submitted version.

## Funding

This work was supported by the National Natural Science Foundation of China (81872686 and 82173611) and the Priority Academic Program for the Development of Jiangsu Higher Education Institutions (Public Health and Preventive Medicine).

## Conflict of interest

The authors declare that the research was conducted in the absence of any commercial or financial relationships that could be construed as a potential conflict of interest.

## Publisher’s note

All claims expressed in this article are solely those of the authors and do not necessarily represent those of their affiliated organizations, or those of the publisher, the editors and the reviewers. Any product that may be evaluated in this article, or claim that may be made by its manufacturer, is not guaranteed or endorsed by the publisher.

## Abbreviations

TC: Total cholesterol ≥ 6.2 mmol/L
TG: Triglyceride ≥ 2.3 mmol/L
LDL-C: Low-density lipoprotein-cholesterol ≥ 4.1 mmol/L
HDL-C: High-density lipoprotein-cholesterol < 1.04 mmol/L.
