# Supplementary material for: SNP rs3803264 polymorphisms in THSD1 and abnormally expressed mRNA are associated with hemorrhagic stroke
Source: Front Aging Neurosci. 2023 Apr 17;15:1144364. doi: 10.3389/fnagi.2023.1144364 (PMC10150931; doi:10.3389/fnagi.2023.1144364)
Supplement: Supplementary file 1 [file Image_1.PDF]

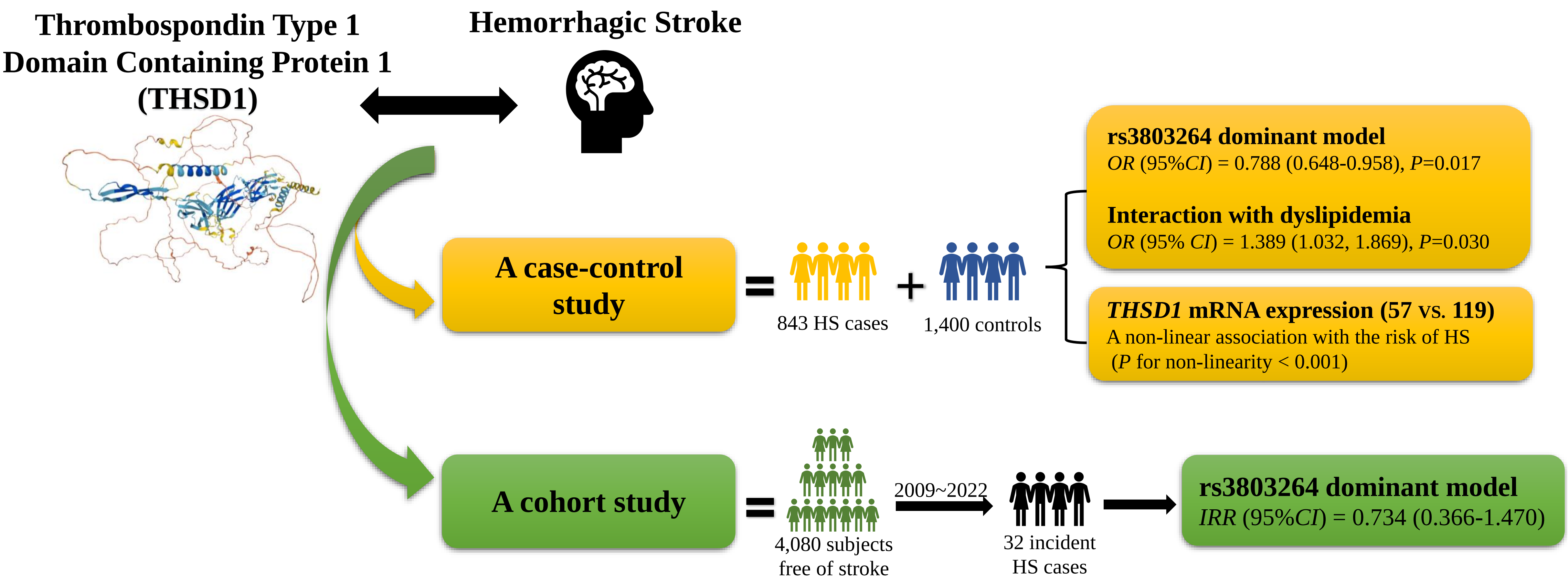

SNP rs3803264 Polymorphisms in *THSD1* and Abnormally Expressed mRNA Are Associated with Hemorrhagic Stroke
