# Supplementary material for: SNP rs3803264 polymorphisms in THSD1 and abnormally expressed mRNA are associated with hemorrhagic stroke
Source: Front Aging Neurosci. 2023 Apr 17;15:1144364. doi: 10.3389/fnagi.2023.1144364 (PMC10150931; doi:10.3389/fnagi.2023.1144364)
Supplement: Supplementary file 2 [file Data_Sheet_1.docx]

***Supplementary materials***

Table S1 Biological information and function prediction for selected tagSNP in *THSD1*

| SNP | Chromosome | Allele | Position | Enhancer | TFBS | eQTL | Nearby Gene | MAF |
| --- | --- | --- | --- | --- | --- | --- | --- | --- |
| rs3803264 | 13 | G/A | synonymous variant | - | - | - | THSD1 | 0.651 |

*SNP, single nucleotide polymorphism; TFBS, transcription factor binding site; eQTL, expression quantitative trait loci; MAF, minor allele frequency.*

Table S2 Demographic and clinical characteristics of the subjects in the cohort study

| Characteristics | Group | non-HS (n=4048) | new HS (n=32) | *Z/χ^2^* | *P* |
| --- | --- | --- | --- | --- | --- |
| Age (year) |  | 58.8 (52.2, 66.9) | 69.0 (60.9, 74.0) | 4.306 | <0.001^a^ |
| Gender [n (%)] | Male | 1633 (40.3) | 20 (62.5) | 6.469 | 0.011^b^ |
|  | Female | 2415 (59.7) | 12 (37.5) |  |  |
| SBP (mmHg) |  | 134 (123, 141) | 137 (127, 145) | 0.943 | 0.346^a^ |
| DBP (mmHg) |  | 82 (78, 89) | 85 (80, 93) | 1.482 | 0.138^a^ |
| GLU (mmol/L) |  | 5.28 (4.85, 5.80) | 5.13 (4.75, 5.46) | 1.627 | 0.104^a^ |
| TC (mmol/L) |  | 4.80 (4.23, 5.46) | 4.47 (3.90, 4.79) | 2.303 | 0.021^a^ |
| TG (mmol/L) |  | 1.32 (0.90, 2.00) | 1.17 (0.70, 2.09) | 0.880 | 0.379^a^ |
| HDL-C (mmol/L) |  | 1.33 (1.13, 1.55) | 1.32 (1.05, 1.70) | 0.106 | 0.915^a^ |
| LDL-C (mmol/L) |  | 2.65 (2.20, 3.12) | 2.37 (1.83, 2.69) | 2.583 | 0.010^a^ |
| Hypertension [n (%)] | Yes | 1959 (48.4) | 22 (68.8) | 5.267 | 0.022^b^ |
|  | No | 2089 (51.6) | 10 (33.2) |  |  |
| Diabetes [n (%)] | Yes | 463 (11.4) | 1 (3.1) | 2.177 | 0.140^b^ |
|  | No | 3585 (88.6) | 31 (96.9) |  |  |
| Dyslipidemia [n (%)] | Yes | 2429 (60.0) | 18 (56.3) | 0.186 | 0.666^b^ |
|  | No | 1619 (40.0) | 14 (43.8) |  |  |

*^a^ Mann-Whitney U test; ^b^ χ^2^ test; HS, hemorrhagic stroke; SBP, systolic blood pressure; DBP, diastolic blood pressure;*

*GLU, glucose; TC, total cholesterol; TG, triglyceride; HDL-C, high density lipoprotein-cholesterol;*

*LDL-C, low density lipoprotein-cholesterol.*

Table S3 Association analyses of rs3803264 and the risk of hypertension and hemorrhagic stroke in the case-control study

| Phenotype | Group | WT/HT/MT | *OR* (95% *CI*), *P* | | |  | Allele | | | *P* for HWE |
| --- | --- | --- | --- | --- | --- | --- | --- | --- | --- | --- |
|  |  |  | Additive model | Dominant model | Recessive model |  | Major/Minor | *OR* (95% *CI*) | *P* ^a^ |  |
| Hypertension | Control | 298/355/96 | Reference | Reference | Reference |  | 0.635/0.365 | Reference | | 0.542 |
|  | Case | 637/675/182 | 0.926 (0.814-1.055), 0.249 | 0.889 (0.197-0.744), 0.197 | 0.944 (0.724-1.229), 0.667 |  | 0.691/0.309 | 0.927 (0.814-1.055) | 0.249 | 0.877 |
| HS | Control | 555/664/181 | Reference | Reference | Reference |  | 0.634/0.366 | Reference | | 0.422 |
|  | HS | 380/366/97 | 0.859 (0.756-0.976), 0.020 | 0.800 (0.673-0.951), 0.012 | 0.876 (0.673-1.139), 0.322 |  | 0.668/0.332 | 0.860 (0.757-0.977) | 0.020 | 0.535 |
|  | ICH | 302/287/75 | 0.847 (0.737-0.973), 0.019 | 0.787 (0.653-0.949), 0.012 | 0.858 (0.644-1.142), 0.293 |  | 0.671/0.329 | 0.848 (0.739-0.973) | 0.019 | 0.586 |
|  | SAH | 78/79/22 | 0.903 (0.716-1.141), 0.394 | 0.850 (0.621-1.164), 0.312 | 0.944 (0.588-1.514), 0.810 |  | 0.656/0.344 | 0.905 (0.718-1.141) | 0.397 | 0.773 |

*WT, wild type; HT, heterozygote; MT, mutant type; HS, hemorrhagic stroke; ICH, intracerebral hemorrhage; SAH, subarachnoid hemorrhage;*

*HWE, Hardy-Weinberg equilibrium. ^a^ P value of χ^2^ test for comparison of allele frequencies between the case and control groups.*

Table S4 Sensitivity analysis for the association of rs3803264 and the risk of hemorrhagic stroke in the case-control study (unadjusted)

| Group | AA/AG/GG | *OR* (95% *CI*), *P* | | |  | *P* for HWE |
| --- | --- | --- | --- | --- | --- | --- |
|  |  | Additive model | Dominant model | Recessive model |  |  |
| Control | 272/356/91 | Reference | Reference | Reference |  | 0.125 |
| HS | 327/324/86 | 0.845 (0.725-0.986), 0.032 | 0.763 (0.619-0.941), 0.011 | 0.912 (0.666-1.249), 0.564 |  | 0.674 |
| ICH | 255/253/67 | 0.845 (0.716-0.996), 0.045 | 0.764 (0.611-0.954), 0.018 | 0.910 (0.650-1.274), 0.584 |  | 0.725 |
| SAH | 72/71/19 | 0.842 (0.649-1.092), 0.194 | 0.761 (0.539-1.074), 0.120 | 0.917 (0.542-1.553), 0.747 |  | 0.815 |

*HS, hemorrhagic stroke; ICH, intracerebral hemorrhage; SAH, subarachnoid hemorrhage; HWE, Hardy-Weinberg equilibrium.*

Table S5 Sensitivity analysis for the association of rs3803264 and the risk of hemorrhagic stroke in the case-control study (adjusted)

| Group | AA/AG/GG | *OR* (95% *CI*), *P* ^a^ | | |  | *P* for HWE |
| --- | --- | --- | --- | --- | --- | --- |
|  |  | Additive model | Dominant model | Recessive model |  |  |
| Control | 272/356/91 | Reference | Reference | Reference |  | 0.125 |
| HS | 327/324/86 | 0.814 (0.680-0.975), 0.026 | 0.757 (0.591-0.970), 0.028 | 0.786 (0.544-1.135), 0.199 |  | 0.674 |
| ICH | 255/253/67 | 0.812 (0.668-0.986), 0.035 | 0.763 (0.584-0.998), 0.048 | 0.761 (0.512-1.129), 0.174 |  | 0.725 |
| SAH | 72/71/19 | 0.772 (0.581-1.025), 0.073 | 0.693 (0.472-1.017), 0.061 | 0.771 (0.435-1.365), 0.372 |  | 0.815 |

*HS, hemorrhagic stroke; ICH, intracerebral hemorrhage; SAH, subarachnoid hemorrhage; HWE, Hardy-Weinberg equilibrium.*

*^a^ Adjusted for age, gender, hypertension, diabetes and dyslipidemia.*

Table S6 Stratification analyses of dyslipidemia for the association of rs3803264 and the risk of hemorrhagic stroke in the cohort study

| Factor | Stratum | Group | AA/AG/GG | *HR* (95% *CI*) | | | | | |
| --- | --- | --- | --- | --- | --- | --- | --- | --- | --- |
|  |  |  |  | Unadjusted dominant model | *I* ^2^ | *P* ^b^ | Adjusted dominant model ^a^ | *I* ^2^ | *P* ^b^ |
| Dyslipidemia | No | non-HS | 685/740/194 | 0.736 (0.258,2.097) | 0.0% | 0.986 | 0.697 (0.245,1.989) | 0.0% | 0.938 |
|  |  | new-HS | 7/5/2 | *P* = 0.566 |  |  | *P* = 0.500 |  |  |
|  | Yes | non-HS | 1020/1078/331 | 0.725 (0.288,1.825) |  |  | 0.744 (0.294,1.885) |  |  |
|  |  | new-HS | 9/8/1 | *P* = 0.494 |  |  | *P* = 0.533 |  |  |
| TC | <5.2mmol/L | non-HS | 1124/1206/313 | 0.864 (0.400,1.868) | 0.0% | 0.400 | 0.819 (0.378,1.776) | 0.0% | 0.463 |
|  |  | new-HS | 12/12/2 | *P* = 0.710 |  |  | *P* = 0.613 |  |  |
|  | ≥5.2mmol/L | non-HS | 581/612/212 | 0.354 (0.065,1.934) |  |  | 0.368 (0.067,2.027) |  |  |
|  |  | new-HS | 4/1/1 | *P* = 0.231 |  |  | *P* = 0.251 |  |  |
| TG | <1.7mmol/L | non-HS | 1133/1195/335 | 1.019 (0.410,2.533) | 0.0% | 0.353 | 0.996 (0.400,2.482) | 0.0% | 0.408 |
|  |  | new-HS | 8/9/2 | *P* = 0.968 |  |  | *P* = 0.993 |  |  |
|  | ≥1.7mmol/L | non-HS | 572/623/190 | 0.441 (0.144,1.349) |  |  | 0.476 (0.155,1.467) |  |  |
|  |  | new-HS | 8/4/1 | *P* = 0.151 |  |  | *P* = 0.196 |  |  |
| HDL-C | ≥1.0mmol/L | non-HS | 1527/1583/471 | 0.930 (0.435,1.987) | 53.4% | 0.143 | 0.938 (0.438,2.011) | 53.7% | 0.142 |
|  |  | new-HS | 12/12/3 | *P* = 0.852 |  |  | *P* = 0.870 |  |  |
|  | <1.0mmol/L | non-HS | 178/235/54 | 0.155 (0.017,1.391) |  |  | 0.155 (0.017,1.391) |  |  |
|  |  | new-HS | 4/1/0 | *P* = 0.096 |  |  | *P* = 0.096 |  |  |
| LDL-C | <3.4mmol/L | non-HS | 1447/1565/430 | 0.778 (0.376,1.612) | 0.0% | 0.709 | 0.767 (0.369,1.592) | 0.0% | 0.690 |
|  |  | new-HS | 14/13/2 | *P* = 0.500 |  |  | *P* = 0.476 |  |  |
|  | ≥3.4mmol/L | non-HS | 258/253/95 | 0.373 (0.034,4.111) |  |  | 0.349 (0.031,3.954) |  |  |
|  |  | new-HS | 2/0/1 | *P* = 0.420 |  |  | *P* = 0.395 |  |  |

^a^ *Adjusted for age, gender,* *smoking, drinking, hypertension, diabetes.* ^b^ *P value for heterogeneity test. HS, hemorrhagic stroke; TC, total cholesterol; TG, triglyceride;*

*HDL-C, high density lipoprotein-cholesterol; LDL-C, low density lipoprotein-cholesterol.*

Table S7 Stratification analyses of rs3803264 genotype for the association of dyslipidemia and the risk of hemorrhagic stroke in the cohort study

| Genotype of rs3803264 | Stratum | Group | non-HS/  new-HS | *HR* (95% *CI*) | | | | | |
| --- | --- | --- | --- | --- | --- | --- | --- | --- | --- |
|  |  |  |  | Unadjusted model | *I* ^2^ | *P* ^b^ | Adjusted model ^a^ | *I* ^2^ | *P* ^b^ |
| AA | Dyslipidemia | No | 685/7 | 0.864 (0.322,2.321) | 0.0% | 0.987 | 0.843 (0.311,2.285) | 0.0% | 0.866 |
|  |  | Yes | 1020/9 | *P* = 0.772 |  |  | *P* = 0.737 |  |  |
| AG+GG | Dyslipidemia | No | 934/7 | 0.852 (0.317,2.287) |  |  | 0.973 (0.357,2.65) |  |  |
|  |  | Yes | 1409/9 | *P* = 0.750 |  |  | *P* = 0.958 |  |  |
| AA | TC | <5.2mmol/L | 1124/12 | 0.647 (0.209,2.005) | 0.0% | 0.478 | 0.592 (0.188,1.866) | 0.0% | 0.520 |
|  |  | ≥5.2mmol/L | 581/4 | *P* = 0.450 |  |  | *P* = 0.371 |  |  |
| AG+GG | TC | <5.2mmol/L | 1519/14 | 0.265 (0.06,1.164) |  |  | 0.262 (0.059,1.166) |  |  |
|  |  | ≥5.2mmol/L | 824/2 | *P* = 0.079 |  |  | *P* = 0.079 |  |  |
| AA | TG | <1.7mmol/L | 1133/8 | 1.972 (0.74,5.253) | 0.0% | 0.382 | 2.153 (0.792,5.847) | 0.0% | 0.444 |
|  |  | ≥1.7mmol/L | 572/8 | *P* = 0.175 |  |  | *P* = 0.133 |  |  |
| AG+GG | TG | <1.7mmol/L | 1530/11 | 0.855 (0.297,2.461) |  |  | 1.036 (0.354,3.026) |  |  |
|  |  | ≥1.7mmol/L | 813/5 | *P* = 0.772 |  |  | *P* = 0.949 |  |  |
| AA | HDL-C | ≥1.0mmol/L | 1527/12 | 2.838 (0.915,8.798) | 13.2% | 0.283 | 2.823 (0.901,8.846) | 0.0% | 0.317 |
|  |  | <1.0mmol/L | 178/4 | *P* = 0.071 |  |  | *P* = 0.075 |  |  |
| AG+GG | HDL-C | ≥1.0mmol/L | 2054/15 | 0.474 (0.063,3.589) |  |  | 0.545 (0.071,4.154) |  |  |
|  |  | <1.0mmol/L | 289/1 | *P* = 0.470 |  |  | *P* = 0.558 |  |  |
| AA | LDL-C | <3.4mmol/L | 1447/14 | 0.801 (0.182,3.526) | 0.0% | 0.710 | 0.748 (0.168,3.325) | 0.0% | 0.719 |
|  |  | ≥3.4mmol/L | 258/2 | *P* = 0.770 |  |  | *P* = 0.703 |  |  |
| AG+GG | LDL-C | <3.4mmol/L | 1995/15 | 0.384 (0.051,2.904) |  |  | 0.365 (0.048,2.776) |  |  |
|  |  | ≥3.4mmol/L | 348/1 | *P* = 0.354 |  |  | *P* = 0.330 |  |  |
| ^a^ *Adjusted for age, gender,* *smoking, drinking, hypertension, diabetes.* ^b^ *P value for heterogeneity test. HS, hemorrhagic stroke; TC, total cholesterol; TG, triglyceride; HDL-C, high density lipoprotein-cholesterol; LDL-C, low density lipoprotein-cholesterol.* | | | | | | | | | |

Table S8 Quantitative trait analyses for SBP, DBP, GLU, TC, TG, HDL-C, LDL-C among genotypes of rs3803264

| Quantitative traits | Genotype | | | | | |
| --- | --- | --- | --- | --- | --- | --- |
|  | AA | AG | GG | *H* | *P*^a^ | *P_trend_* |
| Control | n=555 | n=664 | n=181 |  |  |  |
| SBP (mmHg) | 136 (124, 151) | 135 (124, 149) | 134 (121, 150) | 1.695 | 0.428 | 0.208 |
| DBP (mmHg) | 82 (73, 89) | 81 (74, 89) | 81 (72, 89) | 4.766 | 0.092 | 0.701 |
| GLU (mmol/L) | 5.92 (5.27, 6.10) | 5.93 (5.22, 6.17) | 5.97 (5.28, 6.09) | 0.061 | 0.970 | 0.317 |
| TC (mmol/L) | 5.24 (4.60, 5.82) | 5.13 (4.51, 5.73) | 5.14 (4.41, 5.73) | 2.904 | 0.234 | 0.268 |
| TG (mmol/L) | 1.38 (0.97, 1.97) | 1.35 (0.93, 1.83) | 1.37 (0.98, 1.85) | 1.699 | 0.428 | 0.251 |
| HDL-C (mmol/L) | 1.40 (1.22, 1.57) | 1.40 (1.19, 1.57) | 1.41 (1.19, 1.59) | 0.122 | 0.941 | 0.527 |
| LDL-C (mmol/L) | 2.29 (1.92, 2.86) | 2.29 (1.87, 2.85) | 2.24 (1.87, 2.75) | 1.231 | 0.540 | 0.710 |
| HS | n=380 | n=366 | n=97 |  |  |  |
| SBP (mmHg) | 157 (140, 171) | 150 (136, 170) | 135 (160, 180) | 1.026 | 0.599 | 0.392 |
| DBP (mmHg) | 91 (82, 100) | 90 (80, 100) | 92 (80, 100) | 3.089 | 0.213 | 0.566 |
| GLU (mmol/L) | 6.22 (5.22, 7.51) | 6.41 (5.21, 7.79) | 6.10 (5.09, 7.88) | 0.331 | 0.847 | 0.878 |
| TC (mmol/L) | 4.06 (3.32, 4.73) | 4.02 (3.25, 4.80) | 4.28 (3.46, 4.96) | 3.165 | 0.206 | 0.171 |
| TG (mmol/L) | 1.22 (0.81, 1.75) | 1.19 (0.80, 1.77) | 1.17 (0.81, 1.75) | 0.589 | 0.745 | 0.915 |
| HDL-C (mmol/L) | 1.17 (0.89, 1.35) | 1.11 (0.83, 1.32) | 1.22 (0.86, 1.40) | 4.975 | 0.083 | 0.596 |
| LDL-C (mmol/L) | 2.34 (1.82, 2.78) | 2.29 (1.70, 2.88) | 2.39 (1.77, 2.87) | 0.368 | 0.832 | 0.602 |
| *^a^ Kruskal-Wallis H test; SBP, systolic blood pressure; DBP, diastolic blood pressure; GLU, glucose; TC, total cholesterol; TG, triglyceride; HDL-C, high density lipoprotein-cholesterol; LDL-C, low density lipoprotein-cholesterol; HS, hemorrhagic stroke.* | | | | | | |


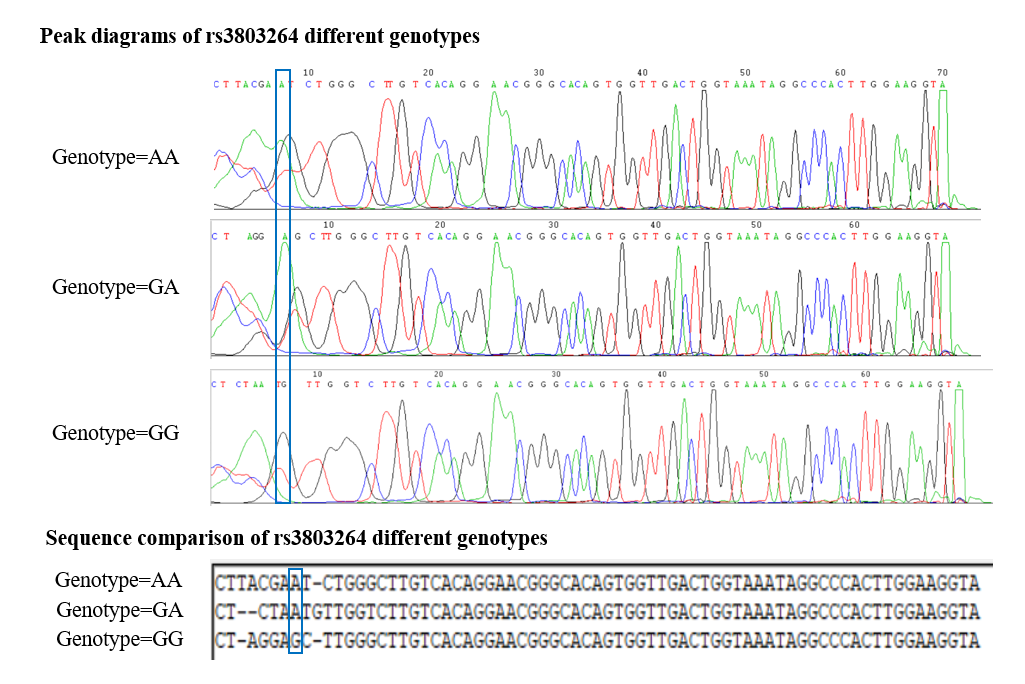


**Figure S1. The sequencing peak diagrams and sequence comparison of rs3803264 different genotypes.** The sequencing results of rs3803264 amplification product are consistent with the genotyping results of TaqMan MGB probe array.


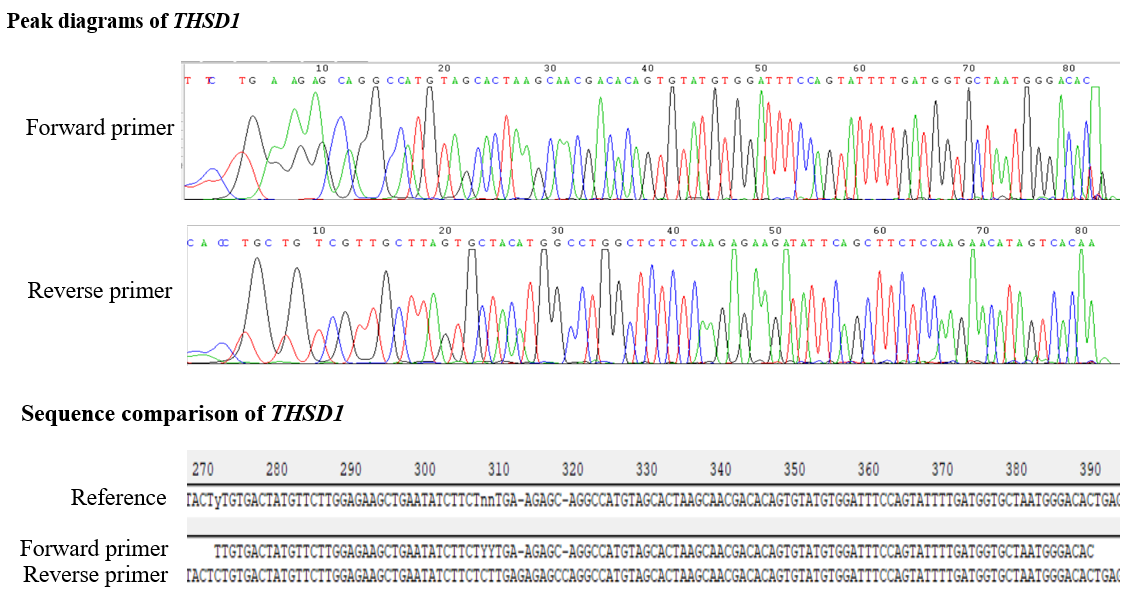


**Figure S2. The sequencing peak diagrams and sequence comparison of *THSD1*.** The length of *THSD1* amplification product is 118bp and the consistency rate of sequence comparison is around 96%.


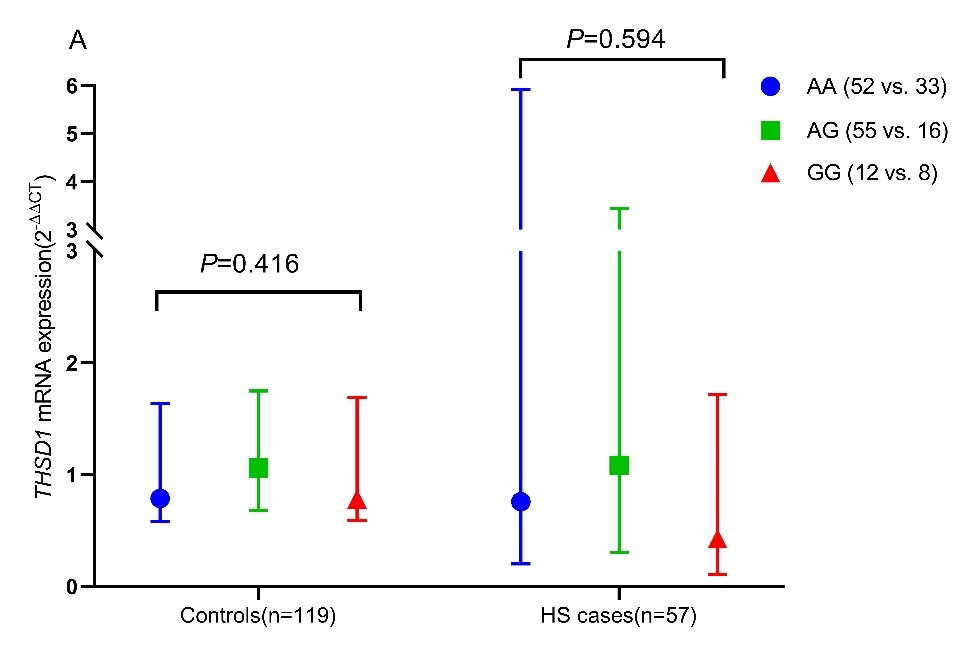

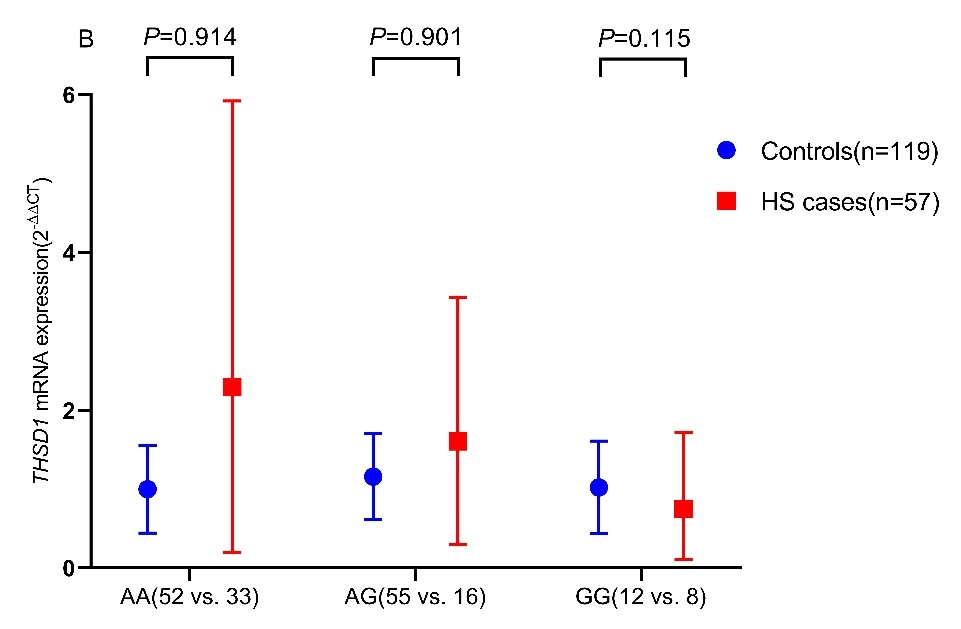


**Figure S3. *THSD1* gene mRNA expression (2^-ΔΔCT^) among different genotypes of rs3803264 and between cases and controls.** There was no significant difference of *THSD1* mRNA expression levels across rs3803264 AA (0.79), AG (1.06), and GG carriers (0.78) in the control group (*P*=0.416) or in the HS cases (0.76, 1.08, 0.43; *P*=0.594). Additionally, there was no significant difference of *THSD1* mRNA expression level between HS cases and controls among the AA (*P*=0.914), AG (*P*=0.901) and GG (*P*=0.115) genotype of rs3803264.
